# Extent of misuse and dependence of codeine-containing products among medical and pharmacy students in a Nigerian University

**DOI:** 10.1186/s12889-019-8074-5

**Published:** 2019-12-19

**Authors:** Wuraola Akande-Sholabi, Rasaq Adisa, Olayinka S. Ilesanmi, Ayomide E. Bello

**Affiliations:** 1 Department of Clinical Pharmacy and Pharmacy Administration, Faculty of Pharmacy, University of Ibadan, Ibadan, Nigeria; 2 Department of Community Medicine, College of Medicine, University of Ibadan, Ibadan, Nigeria

**Keywords:** Codeine-containing products, Opioids misuse, Dependency, Medical and pharmacy students

## Abstract

**Background:**

Misuse and dependency of opioids especially codeine-containing products is of increasing global concern. Inappropriate use of opioids among healthcare students could affect quality of service and ethical conducts of these future professionals, thereby putting the society at risk. This study aimed to evaluate knowledge and perception of medical and pharmacy students in a Nigerian tertiary University on use of opioids with focus on codeine-containing products.

**Methods:**

A cross-sectional survey among 335-medical and 185-pharmacy students from University of Ibadan, Nigeria, between September and December 2018, using a self-administered semi-structured questionnaire.

**Results:**

A total of 178 (34.2%) in multiple responses had used opioid-containing products among the respondents, of this, 171 (96.1%) used codeine-containing formulation. Precisely, 146 (28.1%) of the students had used codeine-containing products before, of this, 16 (11.0%) used the products for non-medical or recreational purpose regarded as a misuse/abuse. In all, 201 (38.7%) had good knowledge of opioid use, with 51 (34.9%) among those who had used opioids and 150 (40.1%) among those who had not used opioids (X^2^ = 1.186; *p* = 0.276). Majority (469; 90.2%) had good perception of risks associated with opioid use; comprising (130; 89.0%) among those who had taken opioids and (339; 90.6%) among those who had not taken opioids before (X^2^ = 0.304; *p* = 0.508). Logistic-regression shows that students who experienced some side effects to be experienced again 22.1 [AOR = 22.1, 95% CI: (5.98–81.72)] as well as those pressured into using codeine-containing products 10.6 [AOR = 10.6, 95% CI: (1.36–82.39)] had more tendency of misuse.

**Conclusion:**

There is a potential for misuse of codeine-containing products among medical and pharmacy students. Peer-influence and experience of some side effects are possible predictors of misuse among the students. Thus, healthcare students’ curriculum should incorporate preventive programme, while public education and policy that favours peer-support programme on medication misuse is advocated for healthcare students.

## Background

Globally, the incidence of misuse of medicines ranges from 4.7 to 67% among students and the abuse of codeine is not an exception [1–9]. Misuse and abuse of medicines is not just an unhealthy practice but a major problem to the society. The misuse and abuse of codeine-containing products is a foremost emerging health challenges in various nations around the globe, this might be because such products are accessible in the range of over-the-counter (OTC) medications which are constantly bought without the need of a doctor’s prescription [10]. In Nigeria, thousands of youths are addicted to codeine-containing products, especially the cough syrup formulation [11, 12]. This is evident by the documentary titled “Sweet Sweet Codeine” released by British Broadcasting Corporation (BBC) in May 2018 [11]. This however, led to the subsequent ban on the importation and sale of codeine as an active pharmaceutical ingredient (API) by the National Agency for Food and Drug Administration and Control (NAFDAC) in Nigeria [12]. The ban on the importation and sale of codeine as an API has led to a drastic scarcity of codeine-containing products within the country, nonetheless, there is presently no legal backing on obtaining these products as a prescription only medicine. As a result, pharmacists are now more cautious about the sale of these products to the public.

Misuse of medicines is defined as the use of drugs with or without prescription, usually outside the acceptable medical practice or medical guidelines. It includes self-medicating with drugs for a longer period, usually at larger doses and for recreational reasons. Medicine misuse is also considered as a problematic consumption where risks and adverse consequences outweigh the benefits [13–15]. The epidemic of opioid abuse has led to significant increase in concern by the global public health authorities and medicines regulatory bodies due to its overall consequences on the youths and society at large [16, 17].

Lifestyle of healthcare professionals play an essential role on the quality of services they render to their patients with a net effect on the society itself [18]. Misuse of medication by the healthcare professionals could also affect their professional conduct and possibly putting the public at a risk [19]. It may therefore be imperative to evaluate healthcare students’ who are the future healthcare professionals on medication misuse.

Marijuana, codeine and alcohol are the top three drugs mostly abused in Nigeria, particularly among teenagers and young adults aged 15–29 years [20]. Codeine (3-methylmorphine), a weak opiate is one of the most widely accessible and commonly used opiates worldwide, particularly because of its analgesic, antitussive and anti-diarrhoeal properties [21]. Although, codeine is considered a weak opiate, drug dependence and side effects such as sedation, euphoria and constipation among others are inherent [15, 22]. In Nigeria and some other developing countries, studies that evaluate extent of use and misuse of opioid-containing substance among youths is scarce. Thus, a need for this study, which assessed the knowledge and perception of codeine-containing products among medical and pharmacy students including their opinion on the risks associated with the use of codeine-containing products. This will provide better understanding with a view to identify areas of contribution for future preventive programme and public education among the healthcare students.

## Methods

### Study design and setting

A cross-sectional study conducted among 335 medical and 185 pharmacy students from the University of Ibadan, Nigeria, between September and December 2018. University of Ibadan is the premier University in Nigeria, with most of the courses as old as the University. In Nigeria, Bachelor of Pharmacy degree is a 5-year programme, while Bachelor of Medicine and Surgery degree is a 6-year programme.

### Sample size determination

Sample size was estimated using Raosoft® sample size calculator (http://www.raosoft.com/samplesize.html), at 95% confidence interval and 5% margin of error, while considering a 50% non-response rate. Based on the assumptions, a target sample size of 297 was calculated. However, considering the nature of the study among students’ population with possibility of higher non-response rate. An attrition rate of close to 100% that will allow for a large sample size was decided.

### Inclusion and exclusion criteria

All consenting male and female students in the first, second, third and fifth year of the Faculty of Pharmacy and College of Medicine, University of Ibadan were included. Pharmacy students in the fourth year, as well as the fourth and sixth year medical students who were on professional experiential assignment during the period of study were excluded.

### Sampling technique

Participants were consecutively approached from each level in the respective faculty, with total sampling of all consented students done, until a target sample population of approximately 590 students was obtained.

### Data collection procedure

At each level in each faculty, a compulsory course for the students was identified. Students were approached shortly after the end of the course, briefed on the objectives and purpose of the study, and subsequently administered the questionnaire. Questionnaire distribution continued every day of the week from each faculty by the principal investigators. Participants were assured of their anonymity and confidentiality of response. Each questionnaire took about 20 to 25 min to be completed, after which the questionnaire was returned and checked for completeness. Measures were put in place to ensure that no student filled more than one questionnaire. This was achieved by coding each questionnaire administered to the students in each faculty to avoid duplication.

### Data collection instrument

The questionnaire for the study was designed and developed by the investigators following extensive review of relevant studies [1, 3, 9] and previous practice experience. The pre-tested semi-structured questionnaire comprised four sections. Section A clarified the demographic information. Section B contained questions that explore students’ knowledge on the use of opioid-containing products, most especially codeine with respect to category of people who can or cannot use opioid, as well as whether opioid use can lead to dependence/addiction among others. Section C consisted of four items that explore the feelings of the students after the use of opioids/codeine-containing products, such as the experience of some effects with the codeine usage which the students would like to experience again, as well as pressured into using codeine-containing products among others. Section D contained 9-item questions with 5-points Likert scale response option ranging from strongly agree (5) to strongly disagree (1) to explore and evaluate students’ perception of the risks associated with the use of codeine-containing products. Examples of such questions included the opinion on whether codeine/codeine-containing products can be abused, as well as whether codeine abuse/misuse can affect psychological well-being.

### Pretest/validation of questionnaire

The questionnaire was assessed for content validity by two academic scholars with public health expertise. This is to ascertain the comprehensiveness of the question-items *vis-a-vis* the study objectives, as well as to ensure that there are no ambiguous questions or statements. A pretest of the questionnaire was also done among 40 randomly selected students from another faculty which was not part of the main study, this is to ascertain the ease of comprehension of the question-items by the would-be respondents, as well as the appropriateness of sampling procedure. Feedback from the pretest and validity assessment led to minor modifications including some questions initially designed in open-ended format which were subsequently re-modified as a dichotomous Yes/No format to eliminate response ambiguity.

### Statistical analysis

Data were coded, sorted, and analysed using SPSS (version 23). Descriptive statistics including frequencies and percentages were used to summarise the data. For the knowledge questions, a correct answer was assigned “one” and incorrect answer was given “zero” Of the 5-knowledge questions, a score of at least four out of the five maximum obtainable score, (i.e. ≥ 80%) was categorised as “good” knowledge, while score <  4 (i.e. < 80%) was categorised as “poor” knowledge. For the 9-item statements on perception of risks with 5-points Likert scale response, a total score of at least 36 (i.e. ≥ 80%) out of the maximum obtainable score of 45 was categorised as “good” perception, while a score <  36 (i.e. < 80%) was assigned “poor” perception of risks. The binary categorisation of scores in the knowledge and perception domains developed for this study was adapted from Bloom’s cut-off criteria, as well as other related studies [23, 24]. Chi-square test was used to evaluate the association between demographic characteristics and those who had used or not use opioid-containing products, as well as the overall knowledge and perception scores.

Misuse/abuse of opioids/codeine-containing products in our study was considered as those who use the products for recreational purposes rather than for intended medical purpose. Three questions related to non-medical use including usage of the product on account of experience/feeling of some effects they would like to experience again, were asked to determine the perceived reasons for abusing opioids-containing products. The three questions served as predictor variables, with binary logistic regression used to evaluate association between the predictors and misuse/abuse of opioid/codeine-containing products. The regression model was estimated using block entry of variables, with all the three variables found significant at a preliminary *p*-value of 10%. The crude logistic regression (unadjusted odds ratio) and adjusted logistic regression (adjusted odds ratio) were subsequently considered. The three explanatory/predictor variables were entered together as one block in the model to represent both the explanatory and cofounders, i.e. the cofounders were not adjusted for separately. The level of statistical significance was set at *p* < 0.05.

## Results

Of the 590 copies of questionnaire administered to the students, 520 were completely filled, given a response rate of 88.1%. Two hundred and sixty-three (50.6%) were male. Seventy-three (50.0%) each of the male and female students had used opioids-containing products, 333 (64.0%) were aged 20 years and above, with a mean age of 20.7 ± 2.8 years. Majority (103; 70.5%) of the students in age group of 20 years and above had used opioids, while 43 (29.5%) used opioids among those aged 16–20 years. Year 3 and year 5 students constituted those who had largely used opioids, 55 (37.7%) and 50 (34.2%), respectively, with statistically significance difference between year of study and user or non-user of opioids (X^2^ = 9.092; *p* = 0.003). Medicine and surgery students were 335 (64.4%), while pharmacy students constituted, 185 (35.6%) among the respondents. A total of 178 (34.2%) in multiple responses had generally used opioid-containing products among the respondents, of this, 171 (96.1%) used codeine-containing formulation, comprising 163 (95.3%) as cough syrups and 8 (4.7%) as non-cough syrup (Table 1).

**Table 1:** Opioids-containing products used by respondents and distribution of demographic characteristics among opioids user and non-opioids users (*n* = 520)

Variable	Using opioids n (%) *n* = 146	Not using opioids n (%) *n* = 374	Chi-square	*p*-value
Gender				
Male	73 (50.0)	190 (50.8)		
Female	73 (50.0)	184 (49.2)	0.027	0.869
Age (year)				
16–20	43 (29.5)	144 (38.5)		
≥ 20	103 (70.5)	230 (61.5)	3.735	0.053
Level of study				
Year 1	5 (3.4)	48 (12.8)		
Year 2	36 (24.7)	101 (27.0)		
Year 3	55 (37.7)	124 (33.2)		
Year 5	50 (34.2)	101 (27.0)	9.092	0.003*
Faculty				
Medicine and surgery	89 (61.0)	246 (65.8)		
Pharmacy	57 (39.0)	128 (34.2)	1.063	0.303
Opioids-containing products used by respondents Ψ (*n* = 178)			n (%)	
Diphenhydramine+dextromethorphan (12.5 mg) with codeine cough syrup (Benylin®)			91 (51.1)	
Ammonium chloride+diphenyhydramine (14 mg) with codeine (10.9 mg) cough syrup (Emzolyn®)			66 (37.1)	
Paracetamol (500 mg) with codeine (8 mg) (Cocodamol®)			8 (4.5)	
Dextromethorphan + diphenhydramine with codeine (9 mg) cough syrup (Myasedyl®)			6 (3.4)	
Hydrocodone			5 (2.8)	
Oxycodone			2 (1.1)	

Table 2 shows the assessment of respondents’ knowledge on use of opioid-containing products. Overall, a total of 201 (38.7%) had good knowledge of opioid use, with 51 (34.9%) among those who had used opioids and 150 (40.1%) among those who had not. Most (95; 65.1%) of the respondents who had used opioids constituted those with poor knowledge compared to those who had not (224; 59.9%), X^2^ = 1.186; *p* = 0.276. Details in Table 2.

**Table 2:** Knowledge on use of opioid-containing products among respondents (n = 520)

	User of opioids-containing products n = 146		Non-user of opioids-containing products n = 374	
Knowledge question	Correct answer n (%)	Wrong answer n (%)	Correct answer n (%)	Wrong answer n (%)
1.Category of people who can use opioids (Answer: Adults and children)	59 (40.4)	87 (59.6)	183 (48.9)	191 (51.1)
2.Category of people who cannot use opioid. (Answer: None of adults and children)	4 (2.7)	142 (97.3)	8 (2.1)	366 (97.9)
3.Can opioid use lead to dependence/addiction (Answer: Yes)	134 (91.8)	12 (8.2)	338 (90.4)	36 (9.6)
4.Addiction is defined as a chronic relaxing brain disease that is characterized by compulsive drug seeking and use despite harmful consequences. (Answer: Yes)	143 (97.9)	3 (2.1)	357 (95.5)	17 (4.5)
5.Drug dependence is a medical term that refers to the state of craving a certain drug in order to function normally. (Answer: Yes)	135 (92.5)	11 (7.5)	344 (92.0)	30 (8.0)
Distribution of scores	Using opioids	Not using opioids		
	n = 146	n = 374		
0	0 (0.0)	3 (100.0)		
1	1 (14.3)	6 (85.7)		
2	13 (37.1)	22 (62.9)		
3	81 (29.6)	193 (70.4)		
4	50 (25.1)	149 (74.9)		
5	1 (50.0)	1 (50.0)		
Cut-off score	Frequency (%)		Remark	
< 4 (i.e. < 80%)	95 (65.1)	224 (59.8)	Poor knowledge	
≥ 4 (i.e. ≥ 80%)	51 (34.9)	150 (40.1)	Good knowledge	
	[X^2^ = 1.186 p-value = 0.276]			

Table 3 shows the respondents’ perception of risks associated with the use of opioid-containing products. A total of 469 (90.2%) had score ≥ 80% indicating good perception of risks associated with opioid use; comprising 130 (89.0%) among those who used opioids and 339 (90.6%) among those who did not, (X^2^ = 0.304; *p* = 0.508). The association between relevant demographic characteristics and respondents’ overall knowledge and perception scores is shown in Table 4. Pharmacy students (87, 47.0%) had significantly good knowledge about opioid usage compared to their medical students’ counterpart (114, 34.0%), X^2^ = 8.490; *p* = 0.004.

**Table 3:** Respondents’ perception on the use of opioids-containing products (n = 520)

Variable	SA	A	U	D	SD	50^th^percentile
	n (%)	n (%)	n (%)	n (%)	n (%)	
1.Codeine/codeine containing products can be abused	399 (76.7)	103 (19.8)	9 (1.7)	3 (0.6)	6 (1.2)	5
2.Codeine abuse/misuse can lead to dependency	397 (76.3)	115 (22.1)	7 (1.6)	0 (0.0)	1 (0.2)	5
3.Codeine abuse/misuse can affect psychological well-being	355 (68.3)	141 (27.1)	19 (3.7)	4 (0.8)	1 (0.2)	5
4.Codeine abuse/misuse can lead to neurological side effects	332 (63.8)	146 (28.1)	37 (7.1)	4 (0.8)	1(0.2)	5
5.Codeine abuse/misuse can lead to socially unacceptable behaviour	336 (64.6)	147 (28.3)	26 (5.0)	11 (2.1)	0 (0.0)	5
6.Codeine abuse/misuse can affect pattern of spending	278 (53.5)	161 (31.0)	66 (12.7)	13 (2.5)	2 (0.4)	5
7.Codeine abuse/misuse can result in accidental overdose	295 (56.7)	185 (35.6)	34 (6.5)	5 (1.0)	1(0.2)	5
8.Codeine abuse/misuse can result in life-threatening withdrawal symptoms	295 (56.7)	185 (35.6)	34 (6.5)	5 (1.0)	1(0.2)	5
9.Codeine abuse/misuse can cause reduced fertility in males and females	129 (24.8)	76 (14.6)	299 (57.5)	16 (3.1)	0 (0.0)	3
Cut-off score	Using opioids (n = 146)		Not using opioids (n = 374)		Remark	
< 36 (i.e. < 80%)	16 (11.0)		35 (9.4)		Poor perception of risks	
≥ 36 (i.e. ≥ 80%)	130 (89.0)		339 (90.6)		Good perception of risks	
	[X^2^ = 0.304 p-value = 0.508]

**Table 4:** Association between relevant demographic characteristics and overall knowledge and perception scores on use of opioids-containing products (*n* = 520)

Variable	Perception of risks, n (%)		Knowledge of use, n (%)	
	Good (score ≥ 80%)	Poor (score < 80%)	Good (score ≥ 80%)	Poor (score < 80%)
Age (years)				
16–20	167 (89.3)	20 (10.7)	88 (47.1)	99 (52.9)
≥ 20	302 (90.7)	31 (9.3)	113 (33.9)	220 (66.1)
	[X^2^ = 0.260 *p*-value = 0.610]		[X^2^ = 8.699 *p*-value = 0.003*]	
Gender				
Male	235 (89.4)	28 (10.6)	93 (35.4)	170 (64.6)
Female	234 (91.1)	23 (8.9)	108 (42.0)	149 (58.0)
	[X^2^ = 0.423 p-value = 0.515]		[X^2^ = 2.433 p-value = 0.119]	
Faculty				
Pharmacy	171 (92.4)	14 (7.6)	87 (47.0)	98 (53.0)
Medicine and surgery	298 (89.0)	37 (11.0)	114 (34.0)	221 (66.0)
	[X^2^ = 1.629 *p*-value = 0.202]		[X^2^ = 8.490 *p*-value = 0.004*]	

*Significance difference with Chi-square (X^2^) test, level of significance, p < 0.05

Table 5 shows the association between the three relevant questions that might serve as predictors of misuse/abuse of opioids/codeine-containing products among respondents. 146 (28.1%) of the students had used codeine-containing products, of this, 16 (11.0%) used codeine-containing products for non-medical or recreational purpose which was considered as a misuse/abuse. The odds of codeine abuse/misuse were 22.1 [AOR = 22.1, 95% CI: (5.98–81.72)] among those who experienced some side effects they would like to experience again. Respondents who were pressured into using codeine-containing products had about 11 times odds of misusing/abusing codeine 10.6 [AOR = 10.6, 95% CI: (1.36–82.39)].

**Table 5:** Predictors of misuse/abuse of opioids-containing products among opioid users (n = 146)

Variable	Misused/abused of opioids/codeine-containing products n (%)		Unadjusted Odds ratio		Adjusted Odds ratio	
			OR (95% CI)	*p*-value	AOR (95% CI)	*p*-value
	Yes	No				
Experienced some side effects I will like to experience again						
Yes	10 (62.5)	12 (9.2)	16.39 (5.07–52.99)	< 0.001	22.1 (5.98–81.72)	< 0.001*
No	6 (37.5)	118 (90.8)	1		1	
Pressured into using codeine-containing product						
Yes	3 (18.8)	4 (3.1)	7.27 (1.47–36.08)	0.015	10.58 (1.36–82.39)	0.024*
No	13 (81.2)	126 (96.9)	1		1	
Need to use regular dose of these products to function well daily						
Yes	2 (12.5)	3 (2.3)	6.05 (0.93–39.33)	0.06	3.42 (0.32–95.46)	0.309
No	14 (87.5)	127 (97.7)	1		1	

*Significant difference with binary logistic regression, level of significance, p < 0.05

Ψ = multiple responses n = number, * significant difference with Chi-square test, level of significance, p < 0.05.

Maximum obtainable score = 5, %individual score = score obtained by an individual ÷ by total obtainable score × 100, X^2^ = Chi-square, level of significance, p < 0.05, n = number.

Exploration of opioid-containing products with focus on codeine as a commonly misuse opioid. Maximum obtainable score = 45; %individual score = score obtained by an individual ÷ by total obtainable score × 100. Strongly agree (SA) = 5, agree (A) = 4, undecided (U) = 3, disagree (D) = 2, strongly disagree (SD) = 1.

## Discussion

Pharmacy studies exploring the misuse of pharmaceutical opioids have concentrated on stronger opioids than weaker opioids like codeine which are regularly available over-the-counter as combination pharmaceuticals products. This study is first in Nigeria conducted among medical and pharmacy students exclusively on codeine use and misuse.

Codeine-containing cough syrup appears to be the most consumed opioid products by the students in their lifetime. Evidence of misuse of these products have been reported in many countries [25–28]. The availability of these products over-the-counter (OTC) without the need of a prescription might explain easy to accessibility with little or no refusal. The intervention of a pharmacist in every sale of OTC codeine-containing product could raise awareness around codeine dependence. The regulation of access to over-the-counter codeine-containing products will assist to identify the patients using these products either for medical purpose or non-medical/misuse. It has been reported that the possibility to buy codeine-containing products from multiple sources, such as; the pharmacy, internet and patent medicine vendors added significantly to the potential for misuse [29]. These discoveries were coherent with other research into over-the-counter medication misuse, with the collective argument being the easy procurement and accessibility [14, 27, 28].

The majority of students in the study had good perceptions of risks about the use of codeine-containing product. This is not surprising as these students are the future healthcare professionals who will be handling these products; thus, they might have been exposed to some information on opioids risks, either in their curriculum or during workshops and seminars. Nonetheless, it was observed that a few of the students precisely 11%, of those, who had previously used these opioid/codeine-containing products before had poor perception of risks about the product. Also, 11 % of students in our study were found to use opioid/codeine-containing products for non-medical purposes at one time or the other in their lifetime, which constitutes a practice considered as misuse/abuse of these products. After adjusting the variables, it was revealed in this study that students who had experienced a side effect they would like to experience again and students who were pressured into using these products were significant predictors for misuse/abuse of these products. These results are similar to findings in Ethiopia and Nepal where peer influence was a significant predictor for misuse among medical and other healthcare students [3, 30]. These results suggest support courses either in the curriculum or as a workshop/seminar could be introduced to decrease inappropriate use of medications including codeine and promote awareness of possible dependence to these medications.

Even though, the perception of risks regarding opioid/codeine-containing products use among respondents was good, it is worthy to note that the knowledge base on the clinical implication with use of these products was unsatisfactory among most of the students. These results are comparable to outcomes in other studies conducted in various parts of the world (1–3, 7). In addition, pharmacy students exhibited better knowledge of use of opioid/codeine-containing products compared to their medical students’ counterparts. This may be expected as medication is the core focus of pharmacists and in the future, they are largely the custodian of medicine. Generally, the poor knowledge about opioid usage among the students could be a pointer to the presence of an educational gap that may need to be filled in healthcare students’ curriculum. This becomes necessary so as to consistently improve students’ knowledge of opioid use with consequent increase in the value-added services to be rendered by this future healthcare providers to the public. This will be especially useful in the context of patients’ counselling on the awareness of addiction and dependence.

### Limitations

This study was conducted among medical and pharmacy students in one university, perhaps if conducted in more universities we might have a more comprehensive scenario of the misuse of codeine-containing products among healthcare students. Also, the exclusion of the fourth and sixth year medical and fourth year pharmacy students, as well as nursing and public health students may not allow for holistic evaluation of all healthcare students in Nigeria. Thus, there may be a need for caution in generalisation of our findings to the entire healthcare students in Nigeria. In addition, subjecting the relatively small proportion of students who previously misused/abused codeine-containing products for non-medical purposes to logistic regression, might have accounted for the relatively large confidence interval observed in our study. Furthermore, the effect of non-consideration of possible confounding factors in the computation of the binary logistics regression, as well as the inherent limitation of self-reported may not be totally excluded. Thus, future study may need to put all these gaps into consideration in order to ensure a far-reaching conclusion in this regard.

## Conclusion

There is a potential for misuse of codeine-containing products among medical and pharmacy students. Peer-influence and experience of some effects are possible predictors of misuse among the students. Thus, healthcare curriculum should incorporate preventive program on medication misuse, while public education and policy that favours peer-support program on medication misuse is advocated for healthcare students.

## Data Availability

The datasets used and/or analysed during the current study are available from the corresponding author on reasonable request.

## Abbreviations

BBC: British Broadcasting Corporation
NAFDAC: National Agency for Food and Drug Administration and Control
OTC: Over the Counter
